# The relationship between uncertainty and trust in genomic medicine and research: A literature review and thematic analysis

**DOI:** 10.1038/s41431-026-02018-9

**Published:** 2026-01-21

**Authors:** Fabian Cannizzo, Lyndsay Newett, Rebekah McWhirter, Ainsley J. Newson, Vanessa Warren, Dianne Nicol

**Affiliations:** 1 Monash University, Melbourne, Australia; 2 Australian National University, Canberra, Australia; 3 University of Sydney, Sydney, Australia; 4 University of Tasmania, Tasmania, Australia

**Keywords:** Genomics, Ethics

## Abstract

The concepts of uncertainty and trust in genomic research and clinical care have not been consistently defined across studies, leading to varied claims about the relationship between them. The role that social groups play in this relationship is also therefore unclear. A categorisation of themes of trust and uncertainty will help to clarify and compare research claims. A review was conducted of peer-reviewed literature that discussed both ‘trust’ and ‘uncertainty’ in genomics research and/or medicine from 1 January 2018 to 28 June 2024. Exclusion criteria removed studies that did not focus on human genomics, and did not mention ‘trust’ or ‘uncertainty’. Discussions of ‘trust’, ‘uncertainty’ and ‘social groups’ were coded into distinct categories. The search returned 1070 unique abstracts from which 26 studies passed the exclusion criteria. Sixteen distinct uses of ‘trust’ and fifteen uses of ‘uncertainty’ were identified alongside sixteen social groups. Relationships between uncertainty and trust were often described as being mediated by a third variable. Irreducible forms of uncertainty reported in studies suggest a need to move towards assisting patients and data donors understand and feel comfortable with uncertainty and make use of ‘productive uncertainties’ to foster trust. More research is needed to understand how social group belonging may shape the relationship between trust and uncertainty.

## Introduction

The success of genomics depends on some degree of public trust in the presence of factual uncertainties [1, 2]. Trust can be broadly defined as ‘an expectation that a person will honour an explicit or implicit commitment’ [3]. In complex social systems, trust becomes a vital tool for patient decision-making. A medical diagnosis may require the participation of dozens of specialists whose work a patient may not have the expertise to fully comprehend. It is therefore imperative to understand how uncertainty in genomics is experienced by patients and research participants, and the effect this might have on their trust in genomics [1]. The purpose of this article is to make a contribution to our understanding of this relationship by reviewing and thematically analysing the literature about uncertainty and trust in genomics.

**Fig. 1:**
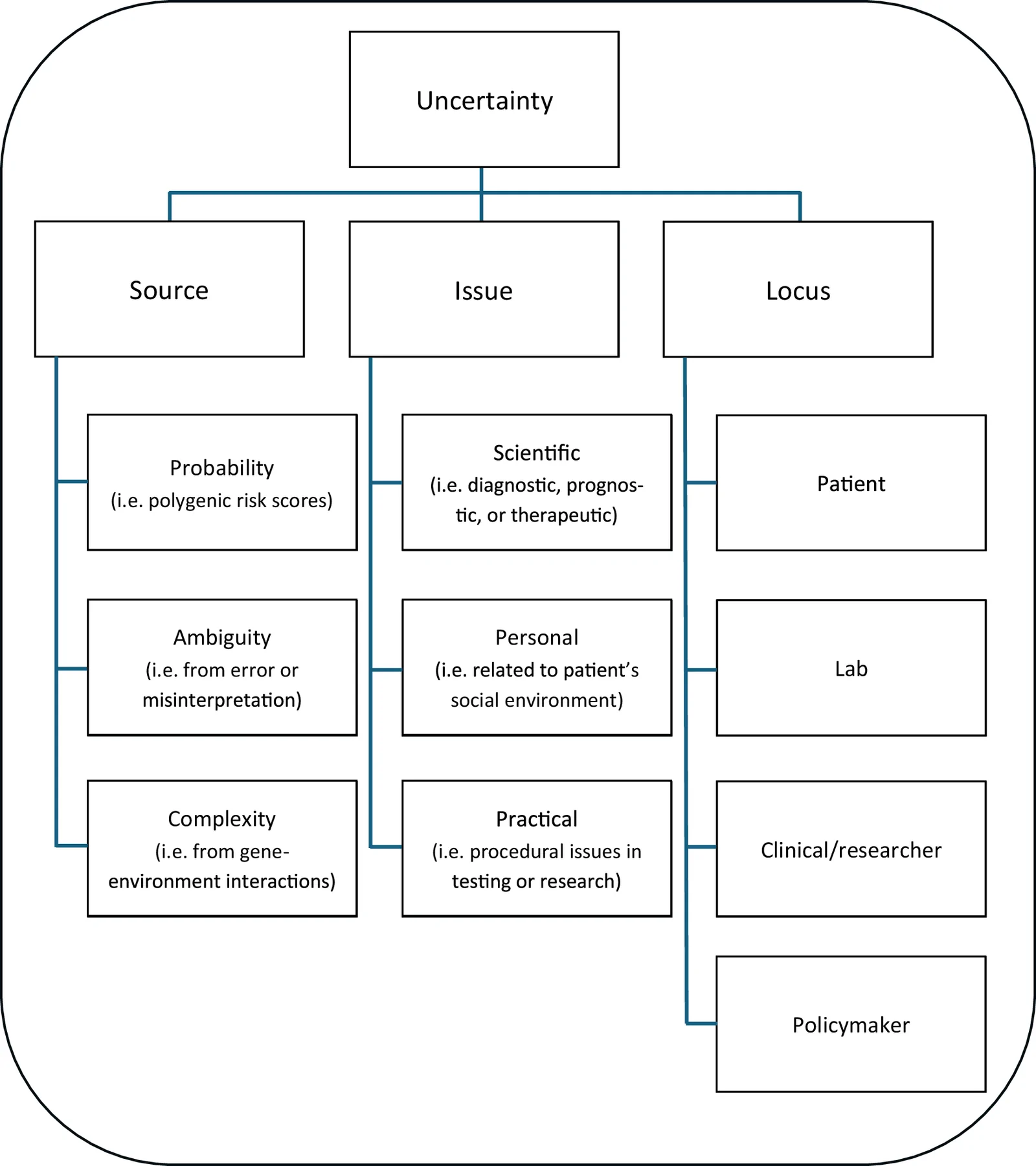
Han et al.’s Taxonomy of Uncertainty. From Newson and Ormond. (55).

Discussions of uncertainty in genomics draw on multiple meanings of the term [4, 5]. In some instances, studies identify uncertainties within scientific practice - such as the extent to which polygenic risk scores may be generalisable across populations - while other papers highlight uncertainties introduced through interpersonal activities such as counselling practices or patient management strategies [1]. To better comprehend how uncertainty plays out in the genomics space, its salient features must be identified. In this regard, Han et al.’s three-dimensional taxonomy of ‘medical uncertainty’ [4] has been influential in studies seeking to categorise uncertainty in genomics [6–9]. Han et al. described medical uncertainty in terms of three dimensions: firstly, the source of uncertainty (sub-divided into forms of probability, ambiguity, and complexity); secondly, the issue of uncertainty (which included scientific, practical and personal varieties); and, thirdly, the locus of uncertainty (which means the person or group who experiences uncertainty) [4]. Figure 1 graphically represents this widely-applied taxonomy. While this taxonomy is useful for clearing up confusion around how the concept of ‘uncertainty’ is used in research, it does not identify how these forms of uncertainty might relate to trust in genomics.

The relationship between trust and uncertainty, and how this affects participation in genomics, has not been consistently conceptualised in the literature. Some assistance is provided in the analysis of this relationship by Samuel et al. [10] Relying on Luhmann’s work on social complexity [11], they describe the connection between trust and uncertainty as ‘the willingness of a trustor to take a risk when uncertainty prevails, based on a subjective belief that a trustee will exhibit reliable behaviour’ [10].

Beyond this, other research suggests that current understandings of the ways these concepts intersect and impact each other, are lacking, and remain unclear. For instance, Bartley et al.’s systematic review indicates that trust in science can lessen the uncertainty that is felt and/or experienced by patients in relation to genomic test results [12]. The impact trust can have on feelings of uncertainty is also highlighted in Griffen et al.’s study in which participants note the importance of having a ‘trustful relationship’ with the doctor who delivers genetic test information [13]. At the same time, Lynch et al.’s study suggests that the effect that trust and uncertainty have on views regarding the return of paediatric research results – and each other – is likely limited [14].

The evidence is both less extensive and more mixed when it comes to the influence of uncertainty on trust. For example, Griffen, Asfahab and Owensa’s exploration of clinicians’ navigation of uncertainty in relation to laboratory reports for whole genome or exome tests suggests uncertainty impacts the trust in laboratory reports by clinicians (or genetic counsellors) who *hold* a level of genetic expertise more than clinicians who do not [15]. Meanwhile, a study assessing the communication of scientific uncertainty – and the impact this can have on public responses to precision medicine research – indicates that communicating uncertainty does not have a consistent effect on trust [16]. Interestingly, in their survey of patients with dilated cardiomyopathy, Ni et al. found that if patients had low levels of trust, they tended to also report low levels of knowledge pertaining to genome sequencing [17]. Although this survey did not specifically explore uncertainty, the relationship between trust and knowledge speaks to the influence that personal (un)certainty may have on trust.

Building trust in genomics requires more specific delineation with regards to what trust is, and the role/s that uncertainties associated with genomics play within trust. We conducted a literature review in order to more clearly define how trust and uncertainty are conceptualised in current genomics literature and synthesise categories for these concepts. We also evaluated the types of relationships between trust and uncertainty identified in studies and assessed which social groups were the focus of those studies. Such an assessment is the first step towards understanding how trust in genomics is impacted by different forms of uncertainty and social group status.

## Materials and methods

### Search methods

An electronic literature search was conducted using Scopus, PubMed and Web of Science for peer-reviewed studies published from 1 January 2018 to 28 June 2024 (see below for a justification of this range). Key terms were divided into three categories and structured so that any returned record must include at least one term from each category. The search terms in the first category included: ‘uncertain’, ‘inconclusive’, and synonyms of these terms. The second category included: ‘genetic’, ‘genomic’, ‘hereditary’, and synonyms. The third category included: ‘trust’, ‘consent’, ‘assurance’, ‘perception’, and synonyms. An example search strategy for PubMed is: (((uncertain*[Title/Abstract] OR inconclusiv*[Title/Abstract]) AND (geneti*[Title/Abstract] OR genom*[Title/Abstract] OR hereditary[Title/Abstract])) AND (trust[Title/Abstract] OR consent*[Title/Abstract] OR assur*[Title/Abstract] OR percei*[Title/Abstract] OR percep*[Title/Abstract])) AND ((“2018/01/01”[Date - Publication] : “2024/06/28”[Date - Publication])).

### Study criteria

#### Study definitions

In this review, the concept of ‘uncertainty’ was defined as an uncertainty related to genomic medicine and research. ‘Trust’ was defined in terms of trust in some entity or phenomenon related to genomic medicine or research. Some existing taxonomies of uncertainty attempt to synthesise past uses of the term by identifying distinct categorical features, as in Han et al.’s categorisation of uncertainty into ‘sources of uncertainty’, the ‘substantive issues’ that underlie uncertainty, and its ‘locus’ in the mind of some individual or another [4]. Other studies limit analysis of uncertainty to particular loci, as in Beisecker’s focus on personal uncertainties experienced by patients with a Lynch Syndrome-related variant of uncertain significance [18]. These categorisations do not consider the relationship between uncertainty and trust and so search parameters were not limited to these existing categorisations. Similarly, the concept of ‘trust’ has been interpreted broadly in this study in order to gather a wide variety of uses of this concept in research.

#### Inclusion criteria

Studies were selected for inclusion if they (1) were written in English; (2) included terms related to both ‘trust’ and ‘uncertainty’; and (3) were published between 1 January 2018 and 28 June 2024. The year 2018 was chosen to capture contemporary research of the impact of genomic uncertainty, given the fast-moving developments within genomic research, and any possible impact of events during the COVID-19 pandemic on public trust towards scientific uncertainty. There were no limits placed on the study design, study population, method of data collection, or country of publication.

#### Exclusion criteria

Studies were excluded if they (i) were not about human genomic research or genomic medicine; (ii) did not discuss uncertainty related to genetics or genomics; and (iii) did not discuss trust in genetics or genomics.

### Study selection method

One author (FC) performed most of the study selection using the following approach: (i) removing clearly irrelevant titles and reviewing abstracts, rejecting those that did not meet the inclusion criteria; (ii) reading full articles of potentially relevant studies to assess whether they fell under the exclusion criteria. A second author (LN) read all abstracts and full articles to assess the selections made by FC, after which any differences in categorisation were discussed and clarified with the whole research team.

### Data extraction and synthesis

Data were produced for individual study characteristics (i.e. study type, aims, methods, summary of results) as well as themes concerning uncertainty, trust and social groups discussed. Social groups were included if the group was a focus of the study or (in the case of a literature review) if the group was the subject of a study reported on in the research paper. Where a randomised sample of a general population was sought, the social group was described as a ‘general public’. Because of the extensive work already performed by Han et al. to produce a taxonomy of forms of medical uncertainty, the categories developed by past researchers were used (although not exclusively) to code uncertainty issues [4, 7]. So, for example, studies that describe examples of probability, ambiguity, or complexity-based uncertainties, as described in Han et al.’s work, were coded, as well as novel or non-systematically defined forms of uncertainty. Similarly, the ‘social groups’ coded in this study are influenced by studies that have described different ‘loci’ of uncertainty, such as patients, participants, family, laboratory personnel, clinicians, investigators, and policy maker [4, 6, 7, 9], but have neither been limited to those categories nor necessarily discovered instances of each of them through coding.

The focus of this literature review was to both thematically categorise uses of ‘trust’ and ‘uncertainty’ in empirical research, as well as to assess whether claims were made about the relationship between these two concepts. Using an inductive content analysis method [19], codes were developed by FC for each of the categories, ‘trust’, ‘uncertainty’, and ‘social group’. RM independently co-coded three of the studies using these coding categories. Final codes were selected through discussion between coders, until a final set of consistent and distinct codes were achieved. Additionally, each study was assessed for any stated relationship between ‘trust’ and ‘uncertainty’. The type of relationship and a summary of the explanation given in the study was recorded.

## Results

### Study selection and characteristics

**Fig. 2:**
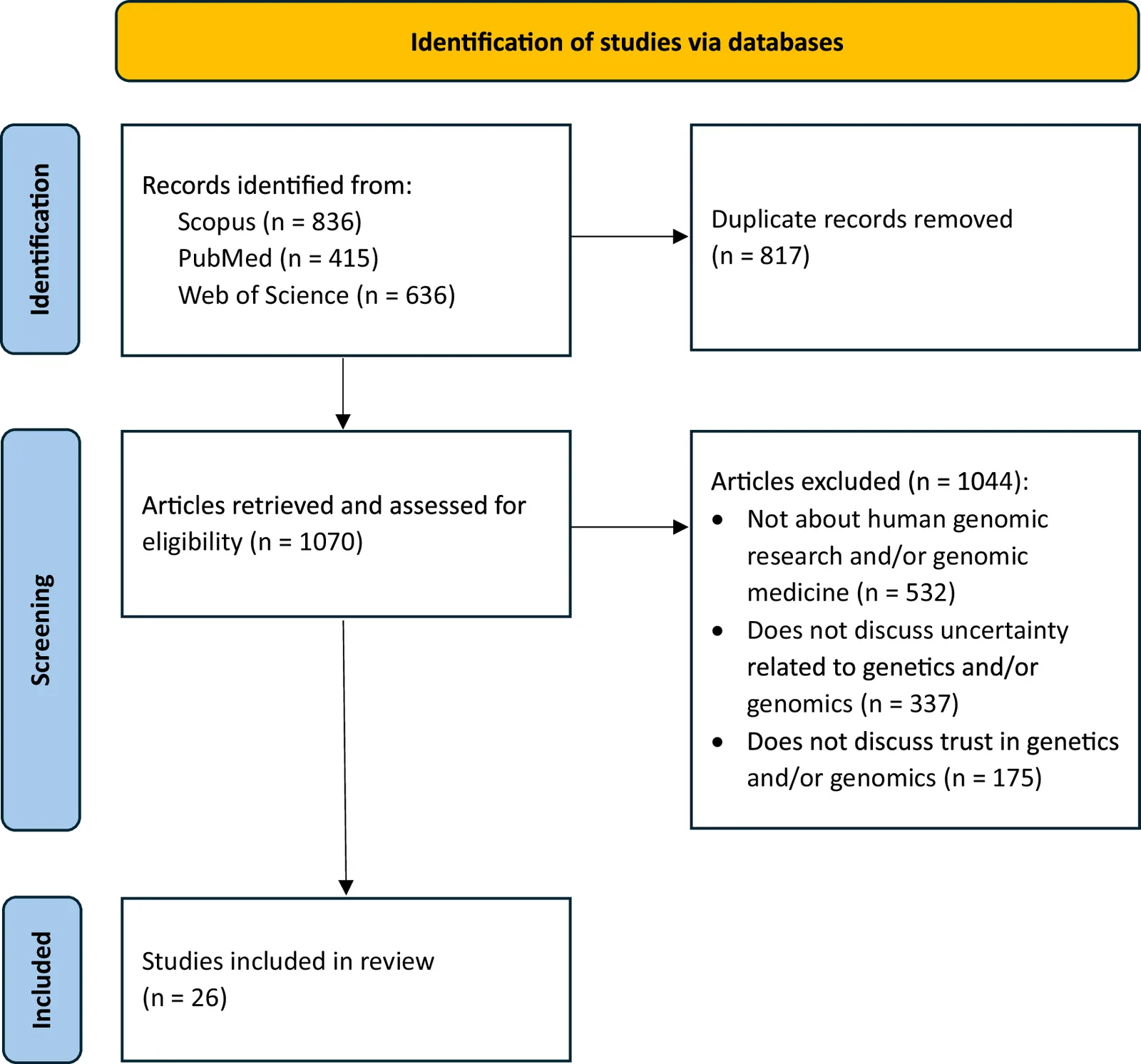
PRIMSA flowchart for literature review and thematic analysis. Inclusion criteria used to assess elegibility were as follows: (1) Descriptions of human genomic research or medical practices, (2) discussion of uncertainty related to genetics and/or genomics, (3) discussion of trust in genomics and/or genomics and (4) articles published in English.

The literature search identified 1070 abstracts after the removal of duplicate records. All abstracts were read and 1044 papers were excluded based on a lack of direct discussion of both trust and uncertainty of some form, or were not about human genomics. The remaining 26 articles that otherwise met the inclusion criteria were included in this review (Fig. 2) [12, 13, 16, 18, 20–42]. Of these articles, 14 (53.8%) were descriptive studies (which aimed to identify and describe phenomena), 6 (23.1%) were analytic papers (which tested hypotheses with experiments), 6 (23%) were reviews of past empirical research and other studies. Table 1 summarises the studies’ characteristics.

**Table 1:** Key characteristics of studies that discussed genomic uncertainty and trust.

1st Author	Year	Study type	Aims	Methods	Summary of results
Anderson	2021	Descriptive	To understand community oncology clinicians’ perceptions of large-panel genomic tumor testing (GTT) and how they relate to clinicians’ intentions to use GTT.	Surveys were used to assess the views and experiences of community oncology physicians in using GTT.	Clinicians had: (1) moderate confidence in their ability to use GTT; (2) lower confidence in patients’ ability to understand test results and access targeted treatment; (3) positive attitudes regarding the value of GTT. Clinicians’ future intentions to use GTT were associated with greater confidence in using GTT and greater perceived barriers to implementing GTT.
Ballard	2020	Descriptive	To garner public views regarding genome sequencing, incidental findings (IFs), and sharing genetic information with relatives.	A survey was distributed to the British general public via a YouGov email.	Respondents had a positive view of genomic sequencing. Around half agreed that their relative had the right to be told about genetic information relevant to them and that they would expect to be told about IFs whether they had asked for them or not.
Bartley	2021	Descriptive	To investigate uncertainty in patients with a cancer of likely hereditary origin while waiting for genome sequencing results.	A questionnaires and an interview were administered at baseline, and at three and 12 months follow up (prior to receiving results)	While participants were motivated to pursue genome sequencing as a strategy to reduce their illness and risk uncertainty, genome sequencing generated additional practical, scientific and personal uncertainties.
Bartley	2022	Descriptive	To understand advanced cancer patients’ experience of uncertainty when receiving comprehensive tumor genomic profiling (CTGP) results, and their perceptions of how healthcare provider (HCP) communication impacts uncertainty.	Semi-structured interviews with advanced cancer patients were conducted within two weeks of patients receiving CTGP results.	Three themes were identified: (1) the type and degree of uncertainty fluctuates with changes in illness and nature of CTGP results; (2) HCPs’ co-ordination of care and communication shapes uncertainty; (3) patients felt that communicating results to reduce relatives’ uncertainty is important.
Biesecker	2019	Review	To describe how clinicians may help patients who receive a return of results indicating variants of unknown clinical significance (VUS) and prepare them for the possibility of their test results indicating VUS.	A narrative review of literature was conducted.	The authors argue that individuals generally experience minimal adverse psychological consequences after learning test results and that clients can be helped to expect uncertain results.
Boumis	2023	Descriptive	To explore BRCA1/2 previvors’ perceptions of the (un)helpfulness of information, what (lack of) barriers previvors face to finding desired information, and the information sources they utilised.	Surveys were conducted with BRCA1/2 previvors about their informational needs, opinions of information, and information sources they utilised.	Previvors generally viewed information as helpful but not always so. They also reported several barriers (including the healthcare system, exclusivity, and uncertainty), while some reported a lack of resistance to finding desired information.
Campbell-Salome	2021	Descriptive	To examine the uncertainty management process of individuals with Lynch Syndrome (LS).	Phone interviews were conducted with individuals with LS, who were asked questions on family communication, risk perceptions, and uncertainty management.	Individuals with LS tried to manage their uncertainty through preventive care, but were often confounded by healthcare barriers. Participants reported increased uncertainty and anxiety due to barriers and used alternative uncertainty management strategies.
Gille	2022	Review	To contribute to reducing uncertainty about future data use and to nurture donor trust for an extended time period.	A narrative review of selective literature with a focus on the authors’ previous works and findings.	The authors conclude that researchers will need to: (1) consider donor autonomy, societal norms and values of the time period in which the data were donated; (2) find mechanisms (where possible) to publicly announce the use of old data sets; and (3) treat the data respectfully.
Grauman	2023	Review	To map out perceptions and concerns regarding how the implementation of precision medicine will impact the doctor-patient relationship in cancer care.	A systematic review of 4 databases was conducted.	The review identified four themes: (1) Providing information addresses issues of information needs and of complex concepts such as genetics and uncertainty; (2) making decisions in a trustful relationship addresses opacity issues and trust; (3) managing negative reactions of non-eligible patients addresses patients’ unmet expectations of precision medicine; and (4) there are issues stemming from physicians’ double role as doctors and researchers.
Halverson	2020	Descriptive	To address a knowledge gap in how patients respond to reclassification of variants of uncertain significance.	Semi-structured interviews were conducted with patients who had received a reclassified genetic test result related to hereditary cancer.	Most of the participants did not express distress related to the variant reclassification; only a minority expressed a decrease in trust in medical genetics; and recall of the new interpretation was limited, even though all participants were recontacted.
Hedgecoe	2023	Descriptive	To explore how groups of professionals make decisions about uncertain aspects of modern clinical genetics/genomics, and how those uncertainties are communicated to patients.	An ethnographic study using data gathered from audio recordings of clinical meetings within a NHS clinical genomics service over a two-year period.	Health care professionals’ perception of, and trust in, the clinical practice at other genomics centres in the NHS, shapes their own application of criteria and the classification of a VUS as either benign or pathogenic.
Horton	2019	Review	To discuss the challenges that the revision of uncertain and complex diagnoses presents for consent and autonomy.	Review article based on selective research.	The authors argue that ‘fully informed’ consent is often not possible in the context of genomic testing, but that an open-ended approach is appropriate.
Makhnoon	2019	Descriptive	To explore the experiences and expectations of patients who received a variant of uncertain significance (VUS) test result and were seeking research studies for variant reclassification.	Semi-structured interviews were conducted with participants with a clinically identified VUS before they enroled into a VUS reclassification study.	Family and personal history of disease were the most prevalent motivators for getting a genetic test. Participants demonstrated mixed understanding of VUS.Some expressed distrust of their providers following a VUS result. Participation in the VUS reclassification study appeared to be motivated by: (1) negative effect about VUS; (2) uncertainty about its impact on clinical management; (3) concern for family members’ well-being; and (4) to advance science.
Makhnoon	2021	Analytic	To examine the potential magnitude of epistemic authority (EA) in response to different variant of uncertain significance (VUS) communication strategies	A factorial experiment was conducted using surveys of adult participants recruited through Amazon Mechanical Turk, where participants were block-randomised to read one of 10 different types of VUS-related scenarios in the context of colon cancer.	GCs were attributed higher EA than gastrointestinal oncologists. Active recommendations were attributed lower EA compared to the inactive. The wrong recommendation was attributed lowest EA compared to the four correct recommendations, which, when dropped from the analysis, showed no difference between the correct active and inactive recommendations.
McMahon	2024	Descriptive	To address a knowledge gap by providing investigator and researcher perspectives on and questions about the assumed the value of return of results (ROR) in precision medicine research (PMR).	Semi-structured interviews with a purposive sample of investigators and researchers across federally funded PMR studies in national consortia, as well as observations of study activities, were conducted.	Some interviewees: (1) validated the value of ROR as a benefit of PMR; (2) questioned the benefit of results for benefitting diverse and disadvantaged populations; (3) expressed uncertainties the benefit of ROR in non-clinical settings; and (4) debated when the promise of the value of ROR may undermine trust in PMR.
Medendorp	2021	Analytic	To examine the relationship between how counsellors convey/address counselees’ uncertainty and councelees’ perceptions of counselling; and to explore the role of individual characteristics on this relationship.	A randomised, controlled experiment was conducted with former counselees, where a video depicting a genetic consultation about multigene panel testing was shown.	The impact of discussing uncertainty on sense of uncertainty, trust in counsellor, and confidence in understanding comprehensive information primarily depends on individual characteristics, such as uncertainty tolerance, trait anxiety and information seeking.
Ratcliff	2023	Analytic	To address a gap in evidence of the effects of disclosing uncertainty in public science messaging, particularly how public audiences process the communication of uncertainty.	A survey was administered to a sample of US adults who were randomly assigned to read one of four versions of a news article in a 2 (certain or uncertain discovery) × 2 (affiliated or unaffiliated scientist attribution) between-participants factorial design.	The uncertainty disclosure had no effect on perceived news article credibility, scientist trustworthiness, objectivity of the scientists’ depiction, or willingness to participate in genomic research. Participants with greater preference for information about uncertainty found the scientists more trustworthy when the research was described as uncertain.
Ratcliff	2021	Analytic	To examine public responses to precision medicine (PM) research, focusing on reactions toward uncertainty about: the scientific impact of sharing data for research, privacy, security, or intended uses of participant data	An online experimental survey was conducted with a sample of US adults who were asked to read a manipulated news article about precision medicine research that conveyed either certainty or uncertainty about PM research.	Conveying uncertainty had no overall effect on outcomes. Those who reported perceiving greater uncertainty had lower attitudes, trust, and willingness to join. Those with more tolerance for uncertainty, support for science, and scientific understanding responded favourably to the scientific uncertainty disclosure.
Raz	2022	Review	To raise awareness of the growing use of next-generation sequencing in public health settings and calls for different models of public engagement and communication about genomics.	Review article based on selective research.	The authors argue that more research is needed to guide communication best practices and propose a research agenda for communication best practices in three contexts: newborn screening, population screening, and largescale DNA biobanks.
Shah	2024	Review	To encourage the widespread adoption of newborn screening sequencing (NBSeq) that would ensure equitable access to precision paediatric health care.	Review article based on selective research.	The authors argue that the feasibility of NBSeq has been demonstrated, but that addressing the challenges facing its widespread adoption requires a multidisciplinary approach involving collaboration among clinicians, researchers, policymakers, ethicists, and stakeholders across various sectors.
Sheikh	2018	Descriptive	To compare how donors in Pakistan and Denmark who deliver material to the same Danish laboratory conceive of ‘trust’ and the nature of their research participation.	Interviews were conducted with people from Pakistan and Denmark who provide blood samples and health data for the same laboratory.	When participants discuss trust they are trying to shape their relationship to researchers while communicating hopes, fears and expectations. The types of trust people talk about involve awareness of uncertainties and risks. There are differences in participants’ trust for researchers in Pakistan and Denmark.
Umstead	2020	Analytic	To explore perceptions of uncertainties in carrier results and how they relate to psychological well-being and health behaviour.	Online and in-person education disclosure sessions were conducted with adult members of a longitudinal sequencing study (ClinSeq) who were carriers for a variant in a gene associated with a disorder inherited in an autosomal recessive pattern who had also not previously received carrier results.	Reductions in affective and evaluative uncertainty followed receipt of results at 1 month. Participants in the web platform arm reported greater clinical uncertainty than those in the genetic counsellor arm at 1 and 6 months. Evaluative uncertainty was associated with a lower likelihood of communicating results to health care providers. Clinical uncertainty was associated with a lower likelihood of communicating results to children.
Underhill-Blazey	2024	Descriptive	To examine how uncertainty manifests in the experience of being at high risk to develop pancreatic cancer and to describe issues, sources of, and responses to uncertainty.	Interviews were conducted with people living with inherited pancreatic cancer risk.	Participants described multiple personal, practical, and scientific issues of uncertainty. They also expressed positive and negative cognitive, emotional, and behavioural responses to uncertainty. Uncertainty sources were both individual experiences and perceptions of healthcare provider uncertainty.
Wöhlke	2019	Descriptive	To analyse lay attitudes and risk perceptions of German lay people on genetic testing with a special focus on how they deal with the numerical information.	Focus groups were conducted with a sample of German people.	Participants showed a positive attitude toward predictive genetic testing. Four discussion topics were identified: (1) Anumeric risk instead of statistical information; (2) Treatment options as a factor for risk evaluation; (3) Epistemic and aleatory uncertainty as moral criticism; (4) Ambivalence as a sign of uncertainty.
Wynn	2018	Descriptive	To explore the process of disclosure of genomic sequencing (GS) results to patients and explore training needs that will help inform the preparation of a larger workforce to manage return of results (RoR)	Semi-structured interviews were conducted with genetic and non-genetic clinicians who delivered RoR for GS.	Reported challenges included managing inflated expectations about GS, returning multiple, unanticipated and/or uncertain results, and navigating a rare diagnosis. Methods to address these challenges included traditional genetic counselling techniques and modifying practice to modulate expectations.
Zhong	2021	Analytic	To examine how features of counsellors’ messaging and message receivers’ intolerance of uncertainty both influenced the effectiveness of genetic counsellors’ (GC) communication of a variant of uncertain significance (VUS) result.	A survey was administered to participants recruited from Amazon Mechanical Turk after they were asked to read a hypothetical conversation with a GC using a 2 (numeric vs. verbal risk format) x 2 (with future plan vs. without future plan) x 2 (GC agency vs. test result agency) between-subject experiment design.	Establishing a future plan and assigning agency to a VUS result enhanced perceived counsellor credibility. When results were presented in a numeric format, assigning agency to counsellors resulted in heightened danger appraisal and greater information-seeking intentions. Individuals’ intolerance of uncertainty moderated the association between risk formats and uncertainty appraisal.

In summary, the studies aimed to: understand patient experience and decision-making (*n* = 10, 38.5%); understand and improve clinical practice (*n* = 8, 30.8%); understand public views of some element of genomics (*n* = 5, 19.2%); understand donor trust in genomics (*n* = 2, 7.7%); or encourage the adoption of genomic sequencing in newborn screening (*n* = 1, 3.8%). Methodological approaches were diverse, including both qualitative approaches, such as interviews (*n* = 9, 34.6%), focus groups (*n* = 1, 3.8%), an ethnographic method (*n* = 1, 3.8%), and quantitative approaches, such as surveys (*n* = 3, 11.5%) and controlled experiments assessed with surveys (*n* = 5, 19.2%), as well as one mixed-methods study that included both surveys and interviews (*n* = 1, 3.8%). We also identified six review studies that included narrative reviews (*n* = 2, 7.7%) and one systematic review (*n* = 1, 3.8%), and three papers that did not articulate a particular methodology (*n* = 3, 11.5%). In their findings, 7 (26.9%) studies presented findings about both ‘trust’ and ‘uncertainty, 10 (38.5%) studies presented findings only about ‘uncertainty’, 3 (11.5%) presented findings only about ‘trust’, and 6 (23.1%) did not present findings directly related to either concept.

### Uses of uncertainty, trust and social groups in research

The initial coding of studies yielded 15 codes for uses of ‘uncertainty’, 16 codes for uses of ‘trust’, and 16 codes for ‘social groups’ (see Table 2). After codes were finalised, a thematic analysis of codes was conducted, and codes were grouped into 5 themes for ‘trust’, 5 for ‘uncertainty’, and 4 for ‘social groups’. The themes for trust are trusting in institutions, losses of trust, experiences of trust, trust as an element of governance, and trust as a research parameter. Themes for uncertainty are informational uncertainty, clinical uncertainty, processual uncertainty, personal uncertainty, and uncertainty as a research parameter. Themes of social groups are data users, medical status groups, database contributors, and social categories.

**Table 2:** Themes of trust, uncertainty and social groups.

Themes	Codes	Number of studies (*n* = 26)	Proportion (%)	Studies (first author name)	
*Uncertainty*					
Informational	Ambiguity and contradictions	6	23	Bartley 2022; Boumis 2023; Halverson 2020; Makhnoon 2019; Umstead 2020; Underhill-Blazey 2024	
	Complexity	6	23	Bartley 2022; Boumis 2023; Medendorp 2021; Umstead 2020; Underhill-Blazey 2024; Wynn 2018	
	Information seeking process	1	4	Boumis 2023	
	Lack of information	3	12	Bartley 2022; Biesecker 2019; Grauman 2023	
	Risk or probability	13	50	Bartley 2021; Bartley 2022; Biesecker 2019; Boumis 2023; Campbell-Salome 2021; Horton 2019; McMahon 2024; Raz 2022; Sheikh 2018; Umstead 2020; Underhill-Blazey 2024; Wöhlke 2019; Zhong 2021	
Clinical/Scientific	Clinical interpretation of results	14	54	Ballard 2020; Bartley 2021; Bartley 2022; Biesecker 2019; Campbell-Salome 2021; Grauman 2023; Hedgecoe 2023; Horton 2019; Makhnoon 2019; McMahon 2024; Medendorp 2021; Umstead 2020; Wynn 2018; Zhong 2021	
	Clinical value of genomic testing	2	8	Anderson 2021; Medendorp 2021	
	Reclassification	2	8	Halverson 2020; Wynn 2018	
	Scientific uncertainty	6	23	Bartley 2022; Grauman 2023; Horton 2019; Ratcliff 2023; Ratcliff 2021; Underhill-Blazey 2024	
	Variant of uncertain significance (VUS)	15	58	Anderson 2021; Bartley 2021; Bartley 2022; Biesecker 2019; Grauman 2023; Halverson 2020; Hedgecoe 2023; Makhnoon 2019; Makhnoon 2021; McMahon 2024; Raz 2022; Shah 2024; Umstead 2020; Wynn 2018; Zhong 2021	
Processual	Future uses of data	3	12	Gille 2022; Horton 2019; Ratcliff 2021	
	Processes for diagnosis or treatment	5	19	Bartley 2021; Bartley 2022; Campbell-Salome 2021; Makhnoon 2019; Underhill-Blazey 2024	
Personal	Personal impact	11	42	Bartley 2021; Bartley 2022; Biesecker 2019; Boumis 2023; Campbell-Salome 2021; Grauman 2023; Halverson 2020; Makhnoon 2019; Umstead 2020; Underhill-Blazey 2024; Zhong 2021	
	Tolerance and resilience	8	31	Bartley 2021; Bartley 2022; Biesecker 2019; Boumis 2023; Campbell-Salome 2021; Medendorp 2021; Ratcliff 2023; Ratcliff 2021	
Research parameter	Uncertainty - operationalisation	8	31	Anderson 2021; Ballard 2020; Bartley 2021; Medendorp 2021; Ratcliff 2023; Ratcliff 2021; Umstead 2020; Zhong 2021	
*Trust*					
Trust in an institution	Biobanks	2	8	Gille 2022; Raz 2022	
	Genomic science, technologies or tests	7	27	Ballard 2020; Halverson 2020; Horton 2019; McMahon 2024; Umstead 2020; Underhill-Blazey 2024; Wöhlke 2019	
	Medical authorities	13	50	Bartley 2021; Bartley 2022; Gille 2022; Grauman 2023; Halverson 2020; Hedgecoe 2023; Makhnoon 2021; Medendorp 2021; Shah 2024; Sheikh 2018; Wöhlke 2019; Wynn 2018; Zhong 2021	
	Private companies	1	4	Sheikh 2018	
	Researchers	5	19	McMahon 2024; Ratcliff 2023; Ratcliff 2021; Raz 2022; Sheikh 2018	
	Systemic or societal	2	8	Gille 2022; Sheikh 2018	
Loss of trust	Medical authorities, loss of trust	9	35	Ballard 2020; Biesecker 2019; Boumis 2023; Campbell-Salome 2021; Grauman 2023; Halverson 2020; Hedgecoe 2023; Makhnoon 2019; Zhong 2021	
	Researchers, loss of trust	1	4	Sheikh 2018	
Experience of trust	Confidence in own understanding of genomics	1	4	Anderson 2021	
	Patient understanding of genomics	1	4	Anderson 2021	
	Religiously mediated	1	4	Sheikh 2018	
	Social interaction	1	4	Gille 2022	
Element of governance	Confidentiality of data subjects	1	4	Ballard 2020	
	Consent as a sign of trust	1	4	Gille 2022	
	Governance for trust	2	8	Gille 2022; Sheikh 2018	
Research parameter	Trust - operationalisation	7	27	Anderson 2021; Gille 2022; Makhnoon 2021; Medendorp 2021; Ratcliff 2023; Ratcliff 2021; Zhong 2021	
*Social groups*					
Data users	Clinical geneticists	2	8	Hedgecoe 2023; Wynn 2018	
	Oncologists	1	4	Anderson 2021	
	Researchers	1	4	McMahon 2024	
Medical status group	Cancer patients	5	19	Bartley 2021; Bartley 2022; Grauman 2023; Medendorp 2021; Underhill-Blazey 2024	
	Previvors	3	12	Boumis 2023; Campbell-Salome 2021; Makhnoon 2019; Medendorp 2021; Underhill-Blazey 2024	
	Patients	2	8	Horton 2019; Umstead 2020	
	Pregnant people	1	4	Biesecker 2019	
	Variant reclassification, patient undergoing	1	4	Halverson 2020	
	VUS, individual with	2	8	Biesecker 2019; Makhnoon 2019	
Database contributor	Donors	1	4	Gille 2022	
	Research participants	1	4	Sheikh 2018	
Social categories	Ethnically diverse populations	2	8	Ratcliff 2021; Shah 2024	
	Indigenous communities	1	4	Raz 2022	
	General public	8	31	Ballard 2020; Makhnoon 2021; Ratcliff 2023; Ratcliff 2021; Raz 2022; Shah 2024; Wöhlke 2019; Zhong 2021	
	Under-represented groups	1	4	McMahon 2024	

### Genomic uncertainty themes

#### Informational

Informational uncertainty refers to uses of, or the absence of, information that results in uneasiness about how to make sense of genomic information. This theme includes five types of uncertainty: (i) encountering seemingly ambiguous or contradictory information; (ii) attempting to understand complex information that may be beyond the scope of one’s personal expertise; (iii) lack of clarity about the appropriate information seeking process; (iv) perceiving a lack of available information; and (v) being presented with risk or probability-based information that may be difficult to use in decision-making. The most common type of information uncertainty is risk or probability-based information, which is described in 13 (50%) of the studies.

#### Clinical/scientific

Clinical/scientific uncertainty refers to genomic uncertainty that manifests in clinical and/or scientific practice. This form of uncertainty is not necessarily due to a lack of access to information that is otherwise available, but more about knowledge of (or disagreement about) known uncertainties in genomic science and clinical care. The following types of uncertainty are included in this theme: (i) the clinical interpretation of genomic test results that include elements of uncertainty; (ii) uncertainty about the clinical value of using genomic testing at all; (iii) the reclassification of a variant of uncertain significance, leading to uncertainty; (iv) scientific uncertainty, such as unknown variables or causes of phenomena; and (v) Variant of Uncertain Significance (VUS) classifications. The two most common types of clinical uncertainty include VUS classifications (*n* = 15, 58%) and uncertainty within the clinical interpretation of results (*n* = 14, 54%).

#### Processual

Processual uncertainty refers to uncertainty stemming from the processes used to manage genomic data use. Two types of processual uncertainty were identified in this review: (i) uncertainty about the future uses of genomic data, which was identified in 3 (12%) studies, and (ii) uncertainty surrounding the processes for diagnosis or treatment of condition identified using genomics, which was identified in 5 (19%) studies.

#### Personal

Personal uncertainty refers to how patients, research participants, or donors of genomic samples and data experience uncertainty related to their genomic test results. This theme included two types of personal uncertainty: (i) descriptions of the personal impact of uncertainty on the decision-making and health of patients/participants/donors, identified in 11 (42%) studies, and (ii) the tolerance and resilience to uncertainty that those groups exhibited, identified in 8 (31%) studies.

#### Research parameter

The concept of uncertainty was also used as a research parameter (or variable) in 8 (31%) of all reviewed studies, where it was operationalised through a research design, as opposed to other studies where the concept was merely discussed.

### Genomic trust themes

#### Trust in an institution

Trust in genomics is described in reviewed studies as being a quality of some institutions. This theme included six types of institutions in which some group held trust relating to genomic data or information: (i) biobanks; (ii) genomic science, technologies or tests; (iii) medical authorities; (iv) private companies; (v) researchers who use genomic data; and (vi) social systems or societies more broadly. The most common type of institutions described were medical authorities, which was identified in 13 (50%) studies, and genomic science, technologies or tests, which was identified in 7 (27%) studies.

#### Loss of trust

Trust is also explicitly described in several studies in terms of a loss of trust in some of the above-named institutions. Two such institutions were identified, with 9 studies coded under this theme referring to a loss of patient trust in medical authorities (35%) and a single study describing a loss of research participant trust in researchers (4%).

#### Experience of trust

Four instances of trust describe how patients, donors, research subjects, or the public experienced trust in genomics. There were four types of experiences of trust identified, each occurring within one (4%) study: (i) confidence in one’s own understanding of genomics; (ii) patients’ understanding of genomics; (iii) the religious mediation of trust-based relationships; and (iv) trust as a form of social interaction.

#### Element of governance

Trust is also described as being an element of the governance of genomic data. Three types of uses of trust in the context of governance include: (i) discussion of the confidentiality of data subjects (*n* = 1, 4%); (ii) interpreting consent as a sign of trust (*n* = 1, 4%); and (iii) governance arrangements to promote trust (*n* = 2, 8%).

#### Research parameter

Trust is also used as a research parameter (or variable) in 7 (27%) studies, where it is operationalised through the research design.

### Social groups addressed

#### Data users

Data users are individuals or groups who access, manipulate, correlate or otherwise transform genomic data or information in order to produce some output. They may have custodial responsibilities and are governed by professional ethics and legal obligations. Three types of data users were identified in reviewed studies: (i) clinical geneticists (*n* = 2, 8%); (ii) oncologists (*n* = 1, 4%); and (iii) researchers (*n* = 1, 4%).

#### Medical status groups

A medical status group is a group of people who share a medical status or common classification, which may be attributed by a medical or research authority. Six types of medical status groups were identified: (i) cancer patients; (ii) previvors; (iii) patients; (iv) pregnant people; (v) patients undergoing the reclassification of a variant of uncertain significance (VUS); and (vi) individuals with a VUS. The most common medical status groups identified in reviewed studies were cancer patients (*n* = 5, 19%) and previvors (*n* = 5, 19%), with most of the latter focusing on people living with Lynch Syndrome [24, 30, 39].

#### Database contributor

Database contributors are individuals whose tissue samples are used to generate genomic data that become part of genomic databases. Two types of database contributors were identified: (i) donors (*n* = 1, 4%) and (ii) research participants (*n* = 1, 4%).

#### Social categories

Social categories are classifications that are applied to numerous persons based on perceived common social characteristics. Four types of social categories were identified in reviewed studies: (i) ethnically diverse populations; (ii) Indigenous communities; (iii) the general public; and (iv) under-represented groups. The most common social categories found in studies is the general public (*n* = 8, 31%).

### Relationship between uncertainty and trust

Each study was assessed to identify how it describes the relationship between trust and uncertainty in genomics. Four types of relationships were identified: causal relationships, referring to claims of cause and effect between one variable and another (*n* = 3, 12%); coincidental relationships, referring to the common co-occurrence of trust and uncertainty (*n* = 7, 27%); mediated relationships, referring to the identification of a third variable that mediates the relationship between trust and uncertainty (*n* = 12, 46%); and no relationships, referring to studies that did not identify any relationship between trust and uncertainty (*n* = 4, 15%). An example of the relationship between trust and uncertainty has been included in Table 3 to illustrate the claims made in these studies.

**Table 3:** Relationship between genomic uncertainty and trust.

1st Author	Year	Relationship*	Explanation
Anderson	2021	Mediated	Uncertainty of the clinical value of genomic tumor testing technologies impacts the trust that oncology physicians have in the technology through the presence of perceived barriers to implementing the technology.
Ballard	2020	Mediated	Uncertainty in the clinical interpretation of genomic data could impact public trust in genomics if unrealistically positive views of genomics entail disappointment and disillusionment with the realities of testing.
Bartley	2021	Coincidental	Cancer patient trusted in medical authorities to understand the clinical significance of their genomic tests results while feeling uncertain about their significance themselves.
Bartley	2022	Causal (Trust as independent variable)	Patient trust in their health care provider’s advice and information led to fewer perceptions of uncertainty about test results and treatment recommendations. Conversely, receiving conflicting information from different healthcare providers (i.e. lower trust in advice) resulted in increased perceptions of uncertainty.
Biesecker	2019	Coincidental	Uncertainties in the information that patients with a Variant of Uncertain Significance receive coincided with discussion of whether results or researchers can be completely trusted.
Boumis	2023	Coincidental	Previvors feel uncertain about whether they are fully informed about their medical status and receiving adequate care. Their trust in doctors is challenged by the perception that providers do not have the latest research and information about their medical condition.
Campbell-Salome	2021	Coincidental	Participants with Lynch Syndrome risks experienced uncertainty around their condition. Some were concerned that their medical care providers had little or no familiarity with Lynch Syndrome and did not trust their judgement.
Gille	2022	Causal (Uncertainty as independent variable)	Uncertainty about future uses of genomic data held by biobanks can threaten donor trust in biobanks.
Grauman	2023	None	Medical professionals can use trusting relationships with their patients to mitigate the negative effects of uncertainty on patient medical care decision-making. Uncertainty is a part of genomics. Trust is a part of medical practice.
Halverson	2020	Mediated	Uncertainty in clinical knowledge, experienced through variant reclassification events, has been linked to patients’ lack of trust in expert practice, although this study found the impact of reclassification on trust was limited.
Hedgecoe	2023	Mediated	When reporting variants of uncertain significance, the failure of medical services to use the same classifications for variants can undermine patients’ confidence in the standards and in medical professionals.
Horton	2019	Causal (Uncertainty as independent variable)	Confidence in the predictive abilities of genomics may be undermined by uncertainties in population studies.
Makhnoon	2019	Mediated	Patients within variant reclassification studies felt distrust towards care providers who they felt were dismissive of their medical concerns.
Makhnoon	2021	Mediated	The impact of uncertainty around variants of uncertain significance on patient’s trust in care providers is mediated by the relationship they have with care providers.
McMahon	2024	Mediated	Uncertainty about the value of return of results can impact the trust research participants have in researchers if participants are given unrealistic expectations about the possible benefits of results for participants.
Medendorp	2021	Mediated	Counselees’ uncertainty tolerance and coping styles moderated how uncertain genomic information impacted their trust in genomic counsellors.
Ratcliff	2023	Mediated	Whether uncertain scientific information is trusted by the general public is mediated by the audience’s information preferences and whether or not scientists reporting the information are affiliated with the research.
Ratcliff	2021	Mediated	The impact of uncertainty disclosure on trust in genomic science is mediated by individual characteristics of participants, such as their general support for science and whether they generally tolerate uncertainty well.
Raz	2022	None	Uncertainty is inherent in genomic research. Public trust is important for the functioning of biobanks. This uncertainty may present an issue for trust, but a precise relationship is not specified.
Shah	2024	None	Uncertainty is inherent in genomic research. Trust is important for integrating genomics into newborn screening programs. No precise relationship between the two is specified.
Sheikh	2018	Coincidental	Genomic research participation involves uncertain risks, so research participants have some degree of trust in researchers.
Umstead	2020	Mediated	The mode of education and result disclosure technique may mediate the relationship between uncertainties experienced by participants and the perceived trustworthiness of results.
Underhill-Blazey	2024	None	Uncertainty is inherent in genomic testing and information. Attaining unexpected results could coincide with mistrust for genomics. No precise relationship between the two is specified.
Wöhlke	2019	Coincidental	Some participants felt uncomfortable with the uncertainty inherent in numerical risk assessments, trusting physicians to make sense of these data.
Wynn	2018	Mediated	Patients’ expectations mediate the relationship between the inherent uncertainty of genomic testing (and variant reclassification) and the trust or mistrust that result from disclosure of genomic information.
Zhong	2021	Coincidental	A genetic counsellor’s communication strategies affect both a patient’s appraisal of uncertainty and perceptions of the counsellor’s credibility.

N.b. The relationships between trust and uncertainty were coded using the following codes: (i) Causal: Proposes a direct cause-effect relationship; (ii) Coincidental: Correlational explanation of phenomena; (iii) Mediated: An intervening variable sits between variables; and (iv) None: No relationship described.

## Discussion

The concepts of uncertainty and trust, as well as the contexts in which they are applied, are inconsistently employed across the literature. Notably, the uses of uncertainty and trust in the publications reviewed here are largely reflective of Han et al.’s taxonomy of medical uncertainty [4]. Complexity, probability and ambiguity (their three identified sources of uncertainty) were very common forms of informational uncertainty, with each category appearing in between 23% and 50% of reviewed studies. Similarly, the three ‘issues of uncertainty’ they propose – scientific uncertainty, practical uncertainty, and personal uncertainty – were common themes in our review, although we have opted to categorise practical uncertainties as being ‘processual’ uncertainties because our subject matter extended beyond the context of ‘structures and processes of care’ [4] and into the context of data management, as noted by the code ‘future uses of data’. While studies such as Han et al.’s have addressed the possible categorisations of inconsistent uses of the concept of uncertainty, no such method has yet been applied to the concept of ‘trust’ in medicine.

Across the articles we reviewed, the interaction between trust and uncertainty has only been outlined in a handful of studies; only three of the studies identified in this review make causal claims about the relationship between trust and uncertainty. What these few studies suggest is that there are plausible causal relationships between trust and uncertainty in both directions, depending on context. Bartley et al. claim that patient trust in their healthcare providers can have an effect on the likelihood with which they perceive uncertainty in genomic test results [22]. In the other direction, Gille and Brall claim that uncertainty about future use of genomic data can impact the trust that people have in biobanks [25], while Horton and Lucassen claim that scientific uncertainties in population studies can undermine general confidence in the predictive abilities of genomics [29]. Notably, the different relational dynamics within clinical and research settings may help to account for some of the differences in focus of these studies: while Bartley et al.’s clinical context paper is concerned with personal uncertainty experienced by patients and factors impacting those experiences, Gille and Brall explore the broader societal values shaping donor trust and biobank participation. Despite these domain-specific differences, the vast majority of surveyed studies described coincidental and mediated relationships, highlighting complex interdependencies between these trust and uncertainty.

To characterise these relationships in a way that renders them of value to our research question, we propose that trust and uncertainty can be categorised by the role that uncertainty plays in offering an opportunity for the actor to demonstrate their trustworthiness. There have been calls for clinicians to accept and engage with patient’s experiences of feeling uncertain and encountering uncertain information, as opposed to focusing on how both kinds of uncertainties can be lessened [6, 8, 9]. The utilisation of mechanisms to reduce uncertainty or to minimise the subjective or disorienting experience of uncertainty – such as the normalisation of uncertain results through counselling, or through reclassification of uncertain results through additional research – is also consistently illustrated [43, 44]. Still, the need to acknowledge and adapt for uncertainty in interactions suggests some genetic counselling approaches help build trust. For example, a ‘counselling approach’, which is focused on providing information in ways that work for the patient (e.g., according to their background, understandings and perceptions), and generating rapport and evidence of empathy, could allow for uncertainty to be better navigated, and for trust to be built [45].

The benefits of altering, or adopting new processes and practices to deal with uncertainty are similarly being highlighted. In a review of guidelines concerning the management of uncertainty in prenatal genomics, Klapwijk et al. identify strategies that are engaged in relation to different kinds of clinical/scientific uncertainty [43]. For instance, the authors suggest that involving a multi-disciplinary team (i.e., different professional staff, including laboratory technicians and clinicians) in decision-making around testing and reporting may work to reduce uncertainty. In fact, when it comes to reporting, or *not* reporting VUS, input from additional stakeholders such as other laboratories, and/or relevant multi-disciplinary committees, is recommended. Meanwhile, others have drawn attention to the need for genomic testing to foster values and qualities such as resilience, welfare, autonomy and solidarity to deal with the cognitive fact of uncertain situations [9].

Focus is additionally placed on the types of strategies that can equip clinicians to better assist patients in managing or accepting personal uncertainty [8, 46]. Key to this is ‘patient-centred’ communication [5], which recognises that both too little and too much information can lead to, or exacerbate, personal uncertainty [5, 12]. The perception of poor patient communication has been suggested to correlate with the reporting of mistrust in healthcare practitioners [45].

Within the context of genetic counselling, emphasis on patient-centred communication can again be understood to promote the use of the counselling approach as described by Kessler [45]. While alternate approaches such as the teaching model centre on information delivery, the counselling approach accounts for personal uncertainty by giving attention to not only the patient’s specific communication requirements, but the outcomes they are seeking from appointments more generally. At the same time, some literature suggests that the timing of information delivery may be significant when it comes to minimising uncertainty. For example, Klapwijk et al. note the importance of preparing patients in pre-test genetic counselling appointments, and via related consent documentation, by explaining the level of uncertainty that could, or that is likely to characterise, or result from, genetic tests. Moreover, the authors signal the benefits of drawing attention to the control patients have in the process, with patients’ ability to opt-out of receiving secondary or incidental findings highlighted. This is perhaps why the development of guidelines that account for the views of all key stakeholders – including, patients – is being promoted to ease experiences of uncertainty in prenatal genomics [43].

Still, because the uncertainties described in studies are often a common or necessary feature of genomics, instead of attempting to minimise uncertainty, it may be necessary to focus on adapting and responding to the events and information that contribute to it. Evidently, reflecting on clinical processes, practices, approaches and information delivery is likely to be beneficial when it comes to navigating both uncertainty and trust in genomic medicine and research. Still, following Newson et al., we argue that an ‘ethics of genomic uncertainty’ is needed to foster trust in the presence of the kinds of uncertain information and situations identified in this review [9]. This may involve clinicians engaging with core ethical concepts such as resilience, welfare, autonomy and solidarity [47, 48], and developing methods for addressing and actioning such in their interactions with patients. For instance, in addition to making patients aware of uncertainties that may arise, offering recommendations and support in relation to how patients can deal with the uncertainty, may be beneficial.

The most common form of informational uncertainty identified in this study, risk and probability, presents a typical case of irreducible uncertainty in both clinical and research genomics. Studies that describe probability or risk-based uncertainties often describe these in negative terms because of the perceived consequences for a patient’s behaviour or emotions: i.e. feeling anxious about genomic test results [12, 24], being reluctant to make healthcare decisions based on risk [23], or becoming overwhelmed by numerical risk information [13]. Similarly, the forms of clinical and scientific uncertainty identified in this review often concern the ordinary functioning of scientific and clinical practice, such as disputes in the interpretation of test results, debate about the value of genomic testing, classification of genetic variants as being of uncertain significance, variant reclassification, and uncertainties surrounding ever developing scientific practices. While communications practices may influence how patients recall or respond to probabilistic information or topics of medical debate [22], it is not clear that the elimination of their awareness of such uncertainties will promote trust, let alone be worthy of trust.

While some of the reviewed studies posit that reducing personal uncertainty should be a goal of genetic counselling and good communication between specialists and laypersons, most studies acknowledge that the relationship between uncertain situations and trust is complex or at least mediated by an intervening variable. Most common among the mediating variables described are the work and strategies that genetic counsellors, researchers, and other public-facing persons use to communicate genomic test results. In such studies, communications strategies often mediate how uncertain outcomes shape trust, such that uncertainty can be a necessary precondition to building trust. In McMahon et al.’s study, for example, the use of appropriate communications strategies could work to foster trust between researchers and study participants in that uncertainty provides an opportunity to demonstrate trustworthiness.

It is also worth noting here the absence of research that socially contextualises how uncertainty shapes trust between communities (such as migrant, sexually or gender diverse, or Aboriginal and Torres Strait Islander) and data custodians. While a single study observed that ‘DNA biobanks and biorepositories need to engage more successfully with indigenous [*sic*] communities’ [35] and two further studies described the need for genomics to engage further with ‘ethnically diverse’ groups [16, 36], none described how genomic uncertainty might be related to trust for peoples in these social contexts. Addressing this literature gap will require the recognition that some forms of genomic uncertainty are precursors to trust between patients/participants and data sponsors (i.e. health services and biobanks). Patients and research participants, although ideally ‘informed’ of their decisions, will necessarily rely on the advice given and promises made by genomic data custodians and their representatives in the presence of uncertain outcomes. Thus, we may describe some forms of uncertainty as ‘productive’ of trusting relationships: a genetic counsellor need not promise certainty to build a trusting relationship with a patient; just as a biobank need not describe every possible research use of genomic data to inspire the trust of donors. Rather, both are trusted to act, to some degree, in the interests of patients and donors. In the case that uncertainty becomes a mechanism for developing trust, we describe these forms of uncertainty as ‘productive uncertainties’.

The case of First Nations peoples is illustrative here. The experiences that First Nation peoples have had, and continue to have, in relation to genomic research and health care, have sown distrust in the field [49]. In Australia, ‘negative past experiences with researchers and other institutional structures’ [50] are considered key to the suspicion and distrust many Aboriginal and Torres Strait Islander peoples feel towards genomics [51]. Distrust of research and medical institutions have also been reported among other First Nations peoples, across North America [52, 53], as well as racial minorities in those contexts [1, 54]. Literature exploring the health inequalities experienced by Indigenous Australians has identified collective vulnerabilities, such as the risk of unauthorised usage of historical DNA samples, uses of genomics to develop genetic definitions of Aboriginality, and uses of genetic analysis that may promote genetic discrimination [55]. The National Centre for Indigenous Genomics, which was formed in 2017 and has an Indigenous-led governance model and Board, is among initiatives to address inequitable healthcare outcomes and uses of genomic data [56]. In developing a model of data governance which requires an Indigenous-led Board to decide how to implement the wishes of data donors, the Centre utilises productive uncertainties. However, such inequities may not be fully accounted for without investigation of how genomic uncertainty may be shaping the participation of Indigenous communities in biobanking, research and healthcare.

### Limitations

This study is limited by the practicalities of conducting a literature review in a field in which technologies, laws and public expectations can transform quickly due to political and economic interest in genomics. While the laws, regulations and sentiments described here may shift with media and political cycles, the more general analysis of the relationship between genomic uncertainty and trust is relevant, if temporally limited.

Additionally, the search terms used in this review reflect the disciplinary and scholarly experiences of the research team at the time of development of the review. However, given the variety of claims already reported here, it does not seem likely that this would have substantially added to our analysis.

### Implications for research and practice

The outcomes of this study have implications for practice and research. Firstly, there is evidence that attention is being paid to the types of uncertainty we identified – specifically, clinical/scientific, informational, and personal uncertainty – with multi-disciplinary input throughout testing and reporting, data sharing, and transparency regarding uncertainty, being emphasised [43]. Still, based on the literature reviewed, processual uncertainty has seemingly received less focus. Additionally, some of the forms of genomic uncertainty described in reviewed studies seem likely irreducible and necessary features of current genomic science. As such, practitioners seeking to foster trust with patients, research participants or donors of genomic samples should not aim to diminish the uncertainties in the eyes of those consenting to genomic testing, perhaps leading to what Corrigan described as an ‘empty ethics’ [57]. Such an approach would not be consistent with common understandings of informed consent. Rather, following Newson’s lead here, it would seem both more prudent and ethically sound to build an ethics of uncertainty into clinical and research practices [9]. In accordance with Newson and Ormond’s articulation of this concept [47], an ethics of uncertainty could be incorporated into genomic counselling and briefings for research participants by drawing on four other core ethical concepts:

- Encouraging *resilience*, through recognition of the impact of uncertainty on those affected and the need to give them time to process and recalibrate;
- Promoting *welfare*, through finding an appropriate balance between too little and too much space for discussing uncertainty;
- Recognising *autonomy*, through provision of a constructive and honest appraisal of uncertainty; and
- Building *solidarity*, through mutual support and partnership.

In the studies reviewed, it is often the case that uncertainty is perceived to correlate negatively with trust in healthcare providers where those uncertainties are not realistically communicated or come as a surprise to patients [21, 25, 29, 32, 42]. The gap between patients’ expectations and counsellors’ desires to retain the confidence of their patients is sometimes described as the result of poor ‘communication strategies’ [42] but seems to also possibly derive from routine uncertainties (such as changing information that may result from variant reclassification) that are beyond the counsellors’ control [27]. In both instances, informing participants about the likelihood of uncertainty persisting and being part of genomic testing – in both pre and post-test appointments [43] – is more conducive to generating ‘productive uncertainties’ than attempting to minimise a patient’s awareness of uncertainty. Furthermore, in addition to focusing on the information being delivered, attention could be given to the way information delivery occurs, with patient-focused approaches – such as the counselling approach [45] – perhaps better equipped to account for the informational and personal uncertainty that can arise in genetic counselling appointments, than others. How to strike a balance between adequately informing patients, and not overburdening them, is beyond the scope of the reviewed studies, but is a worthy task for future research.

A second implication for future research on trust and uncertainty is that studies should be expanded to consider the place of social context and group membership in how genomic uncertainty impacts trust. As noted above, only three studies in this review described Indigenous and ethnically diverse groups, while none described how social group membership might impact on any relationship between trust and uncertainty. Collaborative genetic research projects with Aboriginal communities ‘depend upon strong relationships of trust between the researchers and the communities’ [51], a key step towards enabling Aboriginal communities to benefit from research while minimising harm.

While the participation of Indigenous Peoples and use of Indigenous-led governance have been described as significant steps towards building trust and achieving more just outcomes for communities [50], how uncertainty might shape these practices is not clear. Are the relationships between researchers and communities made more challenging through acknowledgement of uncertainty, or do researchers exercise an ‘ethics of uncertainty’ to build trust? How do Indigenous-led governance groups navigate the uncertainties of genomics in representing the interests of Indigenous individuals and communities? A more direct investigation of trust and uncertainty in these contexts would answer such questions.

## Conclusion

Genomic research, health care, and public health depend upon the trust of patients, donors and communities, who allow custodians to make use of their tissue and data. The role that genomic uncertainty may play in fostering trustworthy relationships between custodians and the subjects of genomic data is important to understand to further develop trustworthy data governance. Our review has highlighted the inconsistent use of these concepts across research literature. Inconsistent uses of concepts may be addressed through articulating whether the uncertainties discussed are an irreducible feature of genomics (as in the case of the possibility of variant reclassifications), or a coincidental problem that can be solved through human action (as in the case of communication strategies). Additionally, the role of social group membership in mediating trust and uncertainty is underexplored and worthy of further research. Australia, as a domain with a diverse population and colonial-settler history, would especially benefit from such research, which could then inform research and policy in countries with similarly diverse populations.

## Data Availability

Additional data are available from the corresponding author on reasonable request.

## References

[1] Atutornu J, Milne R, Costa A, Patch C, Middleton A. Towards equitable and trustworthy genomics research. eBioMedicine. 2022;76:103879. 10.1016/j.ebiom.2022.103879 PMC8850759 35158310

[2] Erlich Y, Williams JB, Glazer D, Yocum K, Farahany N, Olson M, et al. Redefining Genomic Privacy: Trust and Empowerment. Marris C, editor. PLoS Biol. 2014;12:e1001983. 10.1371/journal.pbio.1001983 PMC4219652 25369215

[3] Dive L, Critchley C, Otlowski M, Mason P, Wiersma M, Light E, et al. Public trust and global biobank networks. BMC Med Ethics. 2020;21:73. 10.1186/s12910-020-00515-0 PMC7429755 32799859

[4] Han PKJ, Klein WMP, Arora NK. Varieties of uncertainty in health care: A conceptual taxonomy. Med Decis Making. 2011;31:828–38. 10.1177/0272989X11393976 PMC3146626 22067431

[5] Kerr A, Swallow J, Chekar CK, Cunningham-Burley S. Genomic research and the cancer clinic: uncertainty and expectations in professional accounts. New Genet Soc. 2019;38:222–39. 10.1080/14636778.2019.1586525 PMC6519890 31156350

[6] Biesecker BB, Klein W, Lewis KL, Fisher TC, Wright MF, Biesecker LG, et al. How do research participants perceive “uncertainty” in genome sequencing?. Genet Med. 2014;16:977–80. 10.1038/gim.2014.57 PMC4247806 24875302

[7] Han PKJ, Umstead KL, Bernhardt BA, Green RC, Joffe S, Koenig B, et al. A taxonomy of medical uncertainties in clinical genome sequencing. Genet Med. 2017;19:918–25. 10.1038/gim.2016.212 PMC5517355 28102863

[8] Howard H, Iwarsson E. Mapping uncertainty in genomics. J Risk Res. 2018;21:117–28.

[9] Newson AJ, Leonard SJ, Hall A, Gaff CL. Known unknowns: building an ethics of uncertainty into genomic medicine. BMC Med Genomics. 2016;9:57. 10.1186/s12920-016-0219-0 PMC5009566 27586379

[10] Samuel G, Broekstra R, Gille F, Lucassen A. Public Trust and Trustworthiness in Biobanking: The Need for More Reflexivity. Biopreservat Biobanking. 2022;20:291–6. 10.1089/bio.2021.0109 35172119

[11] Luhmann N, Morgner C, King M Trust and power. English edition. Cambridge, UK; Polity Press; 2017.

[12] Bartley N, Napier C, Butt Z, Schlub T, Best M, Biesecker B, et al. Cancer Patient Experience of Uncertainty While Waiting for Genome Sequencing Results. Front Psychol. 2021;12:1–15. 10.3389/fpsyg.2021.647502 PMC8100530 33967906

[13] Wöhlke S, Schaper M, Schicktanz S. How Uncertainty Influences Lay People’s Attitudes and Risk Perceptions Concerning Predictive Genetic Testing and Risk Communication. Front Genet. 2019;10:380. 10.3389/fgene.2019.00380 PMC6497735 31080458

[14] Lynch J, Hines J, Theodore S, Mitchell M. Lay attitudes toward trust, uncertainty, and the return of pediatric research results in biobanking. AJOB Empir Bioeth. 2016;7:160–6. 10.1080/23294515.2015.1053008 PMC5201190 28050575

[15] Griffen Z, Asfaha DM, Owens K. The Biggest Struggle: Navigating Trust and Uncertainty in Genetic Variant Interpretation. Public Health Genomics. 2024;27:228–32. 10.1159/000542274 PMC11588501 39462497

[16] Ratcliff C, Wong B, Jensen J, Kaphingst K. The Impact of Communicating Uncertainty on Public Responses to Precision Medicine Research. Ann Behav Med. 2021;55:1048–61. 10.1093/abm/kaab050 PMC8557361 34297059

[17] Ni H, Jordan E, Cao J, Kinnamon DD, Gottlieb SS, Hofmeyer M, et al. Knowledge of Genome Sequencing and Trust in Medical Researchers Among Patients of Different Racial and Ethnic Groups With Idiopathic Dilated Cardiomyopathy. JAMA Cardiol. 2023;8:33. 10.1001/jamacardio.2022.4132 PMC9669924 36383367

[18] Biesecker BB, Tibben A, Vos J Uncertainties in genome sequencing. In: Clinical Genome Sequencing: Psychological Considerations [Internet]. 2019. p. 75–88. Available from: https://www.scopus.com/inward/record.uri?eid=2-s2.0-85081905068&doi=10.1016%2fB978-0-12-813335-4.00005-2&partnerID=40&md5=dbb8f97e345cadbe6dba7547441c7f2c.

[19] Vears DF, Gillam L. Inductive content analysis: A guide for beginning qualitative researchers. Focus Health Prof Educ Multi-Prof J. 2022;23:111–27.

[20] Anderson E, Hinton A, Lary C, Fenton A, Antov A, Edelman E, et al. Community oncologists’ perceptions and utilization of large-panel genomic tumor testing. BMC Cancer. 2021;21:1–9. 10.1186/s12885-021-08985-0 PMC8620967 34823486

[21] Ballard L, Horton R, Fenwick A, Lucassen A. Genome sequencing in healthcare: understanding the UK general public’s views and implications for clinical practice. Eur J Hum Genet. 2020;28:155–64. 10.1038/s41431-019-0504-4 PMC6974627 31527856

[22] Bartley N, Best M, Biesecker B, Fisher A, Goldstein D, Meiser B, et al. Effectively communicating comprehensive tumor genomic profiling results: Mitigating uncertainty for advanced cancer patients. Patient Educ Couns. 2022;105:452–9. 10.1016/j.pec.2021.05.018 34016496

[23] Boumis J, Dean M The BRCA1/2 Previvor Information Journey: Understanding What Helps or Hinders. Health Commun. 2023;39:1942–54. 10.1080/10410236.2023.2248677 37622341

[24] Campbell-Salome G, Buchanan A, Hallquist M, Rahm A, Rocha H, Sturm A. Uncertainty management for individuals with Lynch Syndrome: Identifying and responding to healthcare barriers. Patient Educ Couns. 2021;104:403–12. 10.1016/j.pec.2020.07.017 32782180

[25] Gille F, Brall C. Can we know if donor trust expires? About trust relationships and time in the context of open consent for future data use. J Med Ethics. 2022;48:184–8. 10.1136/medethics-2020-106244 33722983

[26] Grauman Å, Ancillotti M, Veldwijk J, Mascalzoni D. Precision cancer medicine and the doctor-patient relationship: a systematic review and narrative synthesis. BMC Med Inform Decis Mak. 2023;23:1–13. 10.1186/s12911-023-02395-x PMC10722840 38098034

[27] Halverson C, Connors L, Wessinger B, Clayton E, Wiesner G. Patient perspectives on variant reclassification after cancer susceptibility testing. Mol Genet Genomic Med. 2020;8:1–8. 10.1002/mgg3.1275 PMC7336756 32329193

[28] Hedgecoe A, Job K, Clarke A. Reflexive standardization and the resolution of uncertainty in the genomics clinic. Soc Stud Sci. 2023;53:358–78. 10.1177/03063127231154863 PMC7614615 36922706

[29] Horton R, Lucassen A. Consent and Autonomy in the Genomics Era. Curr Genet Med Rep. 2019;7:85–91. 10.1007/s40142-019-00164-9 PMC6584705 31281738

[30] Makhnoon S, Garrett L, Burke W, Bowen D, Shirts B. Experiences of patients seeking to participate in variant of uncertain significance reclassification research. J Community Genet. 2019;10:189–96. 10.1007/s12687-018-0375-3 PMC6435771 30027524

[31] Makhnoon S, Mork M, Arun B, Volk R, Peterson S. Perceptions of provider’s epistemic authority in response to variant of uncertain significance-related recommendations. J Genet Couns. 2021;30:513–21. 10.1002/jgc4.1337 PMC8026756 33090616

[32] McMahon CE, Foti N, Jeske M, Britton WR, Fullerton SM, Shim JK, et al. Interrogating the Value of Return of Results for Diverse Populations: Perspectives from Precision Medicine Researchers. AJOB Empir Bioeth. 2024;15:108–19. 10.1080/23294515.2023.2279965 PMC11090989 37962912

[33] Medendorp N, Hillen M, Visser L, Aalfs C, Duijkers F, van Engelen K, et al. A randomized experimental study to test the effects of discussing uncertainty during cancer genetic counseling: different strategies, different outcomes?. Eur J Hum Genet. 2021;29:789–99. 10.1038/s41431-020-00799-1 PMC8110589 33437034

[34] Ratcliff C, Wicke R. How the public evaluates media representations of uncertain science: An integrated explanatory framework. Public Underst Sci. 2023;32:410–27. 10.1177/09636625221122960 36196654

[35] Raz A, Timmermans S, Eyal G, Brothers K, Minari J. Challenges for precision public health communication in the era of genomic medicine. Genet Med. 2022;24:1814–20. Sept. 10.1016/j.gim.2022.05.010 35657379

[36] Shah N, Brlek P, Bulić L, Brenner E, Škaro V, Skelin A, et al. Genomic sequencing for newborn screening: current perspectives and challenges. Croat Med J. 2024;65:261–7. 10.3325/cmj.2024.65.261 PMC11157259 38868972

[37] Sheikh Z, Hoeyer K. “That is why I have trust”: unpacking what ‘trust’ means to participants in international genetic research in Pakistan and Denmark. Med Health Care Philos. 2018;21:169–79. 10.1007/s11019-017-9795-9 PMC5956014 28875227

[38] Umstead K, Han P, Lewis K, Miller I, Hepler C, Thompson L, et al. Perceptions of uncertainties about carrier results identified by exome sequencing in a randomized controlled trial. Transl Behav Med. 2020;10:441–50. 10.1093/tbm/ibz111 PMC7237536 31505002

[39] Underhill-Blazey M, Zhang Y, Stanek S, Norton S. The Experience of Uncertainty in Individuals With High Risk for Pancreatic Cancer. Cancer Nurs. 2024;47:E10–7. 10.1097/NCC.0000000000001171 36480347

[40] Vears DF, Borry P, Savulescu J, Koplin JJ. Old Challenges or New Issues? Genetic Health Professionals’ Experiences Obtaining Informed Consent in Diagnostic Genomic Sequencing. AJOB Empir Bioeth. 2020;12:12–23. 10.1080/23294515.2020.1823906 PMC8120994 33017265

[41] Wynn J, Lewis K, Amendola L, Bernhardt B, Biswas S, Joshi M, et al. Clinical providers’ experiences with returning results from genomic sequencing: an interview study. BMC Med Genomics. 2018;11:1–13. 10.1186/s12920-018-0360-z PMC5941324 29739461

[42] Zhong L, Donovan E, Vangelisti A. Examining the Effectiveness of Genetic Counselors’ Communication of Variant of Uncertain Significance Results of Breast Cancer Genes. Health Commun. 2021;36:606–15. 10.1080/10410236.2020.1733224 32122169

[43] Klapwijk JE, Srebniak MI, Go ATJI, Govaerts LCP, Lewis C, Hammond J, et al. How to deal with uncertainty in prenatal genomics: A systematic review of guidelines and policies. Clin Genet. 2021;100:647–58. 10.1111/cge.14010 PMC8596644 34155632

[44] Kaufman R, Timmermans S, Raz A. Genomic uncertainty and genetic counsellors’ professional authority. Sociol Health Illn. 2023;45:485–502. 10.1111/1467-9566.13582 36424363

[45] Kessler S. Psychological aspects of genetic counseling. IX. Teaching and counseling. J Genetic Counsel. 1997;6:287–95. 10.1023/A:1025676205440 26142236

[46] Fallowfield L, Solis-Trapala I, Starkings R, May S, Matthews L, Eccles D, et al. Talking about Risk, UncertaintieS of Testing IN Genetics (TRUSTING): development and evaluation of an educational programme for healthcare professionals about BRCA1 & BRCA2 testing. Br J CancerANCER. 2022;127:1116–22. 10.1038/s41416-022-01871-x PMC9470577 35715636

[47] Newson AJ, Ormond KE. Genetic Counselling: Genomic Uncertainties. eLS. 2025;4:1–8. 10.1002/9780470015902.a0029669.

[48] Schumann S, Gschmeidler B, Pellegrini G. Knowing, relationships and trust—citizens’ perceptions of whole genome sequencing for the Genetics Clinic of the Future. J Commun Genet. 2021;12:67–80. 10.1007/s12687-020-00486-0 PMC7846639 32997319

[49] Garrison NA, Hudson M, Ballantyne LL, Garba I, Martinez A, Taualii M, et al. Genomic Research Through an Indigenous Lens: Understanding the Expectations. Annu Rev Genomics Hum Genet. 2019;20:495–517. 10.1146/annurev-genom-083118-015434 30892943

[50] Kowal E, Pearson G, Peacock CS, Jamieson SE, Blackwell JM. Genetic Research and Aboriginal and Torres Strait Islander Australians. J Bioethical Inq. 2012;9:419–32. 10.1007/s11673-012-9391-x 23188401

[51] McWhirter R, Nicol D, Savulescu J. Genomics in research and health care with Aboriginal and Torres Strait Islander peoples. Monash Bioeth Rev. 2015;33:203–9. 10.1007/s40592-015-0037-8 26507135

[52] Morgan J, Coe RR, Lesueur R, Kenny R, Price R, Makela N, et al. Indigenous Peoples and genomics: Starting a conversation. J Genet Couns. 2019;28:407–18. 10.1002/jgc4.1073 PMC7379939 30629780

[53] Barton KS, Porter KM, Mai T, Claw KG, Hiratsuka VY, Carroll SR, et al. Genetic research within Indigenous communities: Engagement opportunities and pathways forward. Genet Med. 2024;26:101158. 10.1016/j.gim.2024.101158 38699966

[54] Scherr CL, Ramesh S, Marshall-Fricker C, Perera MA. A Review of African Americans’ Beliefs and Attitudes About Genomic Studies: Opportunities for Message Design. Front Genet. 2019;10:548. 10.3389/fgene.2019.00548 PMC6587098 31258547

[55] Tait B, Carpenter B. Indigenous Australians, genomics, and the law. AlterNative. 2024;20:618–26.

[56] Elsum I, McEwan C, Kowal EE, Cadet-James Y, Kelaher M, Woodward L. Inclusion of Indigenous Australians in biobanks: a step to reducing inequity in health care. Med J Aust. 2019;211:7. 10.5694/mja2.50219 PMC6852395 31155726

[57] Corrigan O. Empty ethics: the problem with informed consent. Sociol Health Illn. 2003;25:768–92. 10.1046/j.1467-9566.2003.00369.x 19780205

